# Adverse Event Reporting in Older Adult Mental Health: A Theme Analysis

**DOI:** 10.1192/bjo.2024.478

**Published:** 2024-08-01

**Authors:** Kim Herbert, Ashley Fergie

**Affiliations:** NHS Greater Glasgow & Clyde, Glasgow, United Kingdom

## Abstract

**Aims:**

We carried out a theme analysis of SAER (Significant Adverse Event) reports completed in Older Adults Mental Health Services in Greater Glasgow & Clyde. We wanted to identify common themes to bring about shared learning.

**Methods:**

We analysed 19 SAERs from 2017 to 2023, using deductive coding. The ‘Human & Contributory Factors’ included within the SAER toolkit formed the coding system. Coding was then discussed between authors to explore the themes.

**Results:**

Considering the demographics of the group, patients who died by suicide demonstrated gender distribution and methods in keeping with recognised statistics. However there was an over-representation of anxiety disorders and grief reaction (64%). This may prompt clinicians to hold a lower threshold for risk management strategies in this group.

*Theme #1: ‘Management & Organisation’*. In the period covering the pandemic, reports reflected the need for rapid changes in practice and how in some cases this had an impact on patient care e.g. restricting the possibility for review in the patient's home.

Challenges in liaising with external agencies such as Police Scotland were also highlighted

Many reports reflected that practice could have been updated, encouraging willingness to scrutinize long-standing practice.

*Theme #2: ‘Communication & Team factors’*. Communication failures between staff were more common than with patients. It was more common for communication failures to occur between teams than within.

This theme also covered issues with availability of information, such as the hybrid model of working with electronic systems but also with some paper records, and the opportunity for information to be missed as a result.

*Theme #3: Quality of Care*. This theme referred to recommendations for more robust or formalized methods of working, or for care to be more clearly patient-centred.

Delays accessing care were also highlighted. This might refer to a delay accessing other treatments within the inpatient setting, or to missed opportunities or delays in outpatient assessment.

*Factors around specific tasks were frequently identified*. Most often this referred to guidelines not being followed (updating formal risk assessments, referral to Tissue Viability, etc.). In a smaller number of reports it was identified that guidance was insufficient with recommendations for these to be reviewed.

The importance of patient factors was acknowledged in all reports without this apportioning blame to them or absolving the team from identifying areas for improvement.

**Conclusion:**

This theme analysis identified a number of key themes for older adult psychiatry teams to consider. Results have been disseminated locally.

